# Milk restriction or oligosaccharide supplementation in calves improves compensatory gain and digestive tract development without changing hormone levels

**DOI:** 10.1371/journal.pone.0214626

**Published:** 2019-03-28

**Authors:** Natália Alves Costa, Aline Priscila Pansani, Carlos Henrique de Castro, Diego Basile Colugnati, Carlos Henrique Xaxier, Katia Cylene Guimarães, Luiza Antas Rabelo, Valéria Nunes-Souza, Luis Fernando Souza Caixeta, Reginaldo Nassar Ferreira

**Affiliations:** 1 Department of Physiological Sciences, Institute of Biological Sciences, Federal University of Goiás, Goiania, Goiás, Brazil; 2 Department of Animal Science, Animal Nutrition Laboratory, Goiano Instituto Federal, Rio Verde, Goiás, Brazil; 3 Department of Physiological Sciences, Institute of Biological Sciences, Federal University of Alagoas, Maceió, Alagoas, Brazil; 4 Department of Physiological and Pharmacology Sciences, Institute of Biological Sciences, Federal University of Pernambuco, Recife, Pernambuco, Brazil; University of Illinois, UNITED STATES

## Abstract

We estimated the effect of oligosaccharide supplementation and feed restriction on calves. The study was divided into two experimental periods of 28 days each with 20 crossbred calves that had initial body weight of 37 Kg and housed in individual pens. The animals were split in four experimental groups: animals fed 6 L milk/day (CON) in the two periods, animals fed milk restricted (3 L milk/day) in the first period and followed by CON feeding in the second period (RES), animals receiving supplementation of 5 g/day of mannanoligosaccharide (MOS) and animals receiving supplementation of 5 g/day mannan and frutoligosaccharide (MFOS). At the end of the study, all the animals were slaughtered. The average weight gain was lower in the restricted group when compared with CON and MFOS groups in the first period (P < 0.05) and there were no difference among the groups in the second period. Animals supplemented with MOS showed a significant increases in jejunal villus height and rumen papillae, which were not observed for MFOS group (P < 0.05) compared with RES and CON groups. There were no difference in ghrelin and leptin levels among treatments during periods 1 and 2 (P > 0.05). Also, the expression of ghrelin receptors in the paraventricular region of the hypothalamus did not differ among groups. We conclude that milk restriction during the first weeks of life in calves resulted in compensatory gain and did not modify the hormonal profile and expression of the ghrelin receptor in the hypothalamus. Moreover, a prebiotic supplementation changed the development of intestinal and ruminal epithelium.

## Introduction

The pre-weaned calf is the most at-risk population of cattle on the farm for gastrointestinal malfunction [1] and the first three months of calves’ life are marked by several physiological and digestive changes [2]. Often at this stage the animals do not adapt to all transformations and modifications in their digestive physiology [3] resulting in high morbidity and mortality. The use of dietary strategies and supplementation of substances that act as growth factors are frequently chosen as a strategy to help overcoming these periods and to improve the maturation and digestive function of the gastrointestinal tract (GIT) [4–6].

Prebiotics are able to modulate the intestinal microbiota and may be a choice while the aim is to improve growth of pre-weaned calves, nutrient absorption and intestinal development [7]. Oligosaccharide is a group of prebiotics well known for boosting the gastrointestinal tract development. Within this group, mannanoligosaccharides (MOS) and fructooligosaccharides (FOS) display good results [8,9]. Previous studies have shown that addition of prebiotics to animal diets can improve mucosal immune system function, average daily gain and fecal scoring [10–12]. Otherwise, other studies show that oligosaccharide supplementation display no significant effects on performance or gastrointestinal development [11, 13,14]. Calves receiving supplementation of 4 g/day of MOS had improvement in body weight (BW) and structural growth measurements were better compared to control group [9]. Prebiotics may also be involved in secretion of the hormone of satiety, delayed gastric emptying and increased energy expenditure [15]. The mechanisms underlying these effects comprise reductions in the release of the orexigenic hormone ghrelin by the endocrine gastrointestinal cells in response to nutritional stimuli [16].

Another alternative to improve intestinal and ruminal development and accelerate the transition to the stage of functional ruminants is the milk restricted feeding for a period, which accelerates the development during the re-alimentation post-restriction period [17]. The dietary restriction promotes a reduction in the requirement for maintenance energy due to the lower body weight with consequent compensatory gain (CG) upon re-alimentation [17,18]. However, the mechanisms controlling the CG are ambiguous and the interpretation of the results from these studies is often confounded due to methodological differences in the age, body weight, timeframe of feed restriction and subsequent re-alimentation, as well as changes in diet composition between restricted and re-alimented periods [19,20]. Therefore, to further understand the mechanisms underlying the CG, it is important to investigate how the molecular aspects of tissues metabolically activates during the CG. This comprehension would lead to a better exploration and a possible incorporation of this approach in the management of beef cattle [21]. The aim of this study was to compare two feeding techniques for suckling calves and verifying metabolic-hormonal changes that could promote better development, growth, weight gain and animal health to prepare calves for weaning period.

## Material and methods

All procedures involving animals were approved by Ethics Committee on Animal Use (CEUA) from Federal University of Goias (UFG)–Protocol 017/16 (S1 File).

### Animals and feeding

Calves were provided as donation by local breeders, from farms located about 50km near to Goiânia–GO, Brazil. Twenty crossbred dairy calves were used in this study, at the beginning of the experiment calves had a mean live weight of 37.83 ± 4.92 Kg. The animals were identified by ear rings and housed individually in twenty covered pens with concrete floors. Cleaning and disinfection of the environment was performed by daily washing using hypochlorite solution (10%). Calves were fed pooled colostrum before their arrival at the experimental site for the first two days of life. The calves went through an adaptation period of four days, receiving in this period 6 L of whole milk/day and *ad libitum* initial grounded concentrate (based on corn, soybean meal and mineral premix), hay and water. The health and behavior of animals were monitored daily before the morning suckling.

Thereafter, the animals were randomly distributed into four different experimental groups, CON, RES, MOS and MFOS with 5 animals in each. CON calves received 6 L of milk/day for 56 days. RES calves received restriction milk (3 L/day) for 28 days (Period 1) followed by 6 L milk/day for 28 days (Period 2). MOS animals had supplementation of 5 g/day of mannanoligosaccharides (GlucanMos–Yes, Brazil) and same diet of CON animals. MOS calves had supplementation of 5 g/day of manan-fructooligosaccharides (GlucanMosFos–Yes, Brazil) and same diet of CON animals. Calves had ad libitum access to water, tifton hay and the starter grain diet (based on corn, soybean meal and mineral premix), which met the requirements for pre-weaned calves throughout the study. The milk was offered twice a day, at 8:00 h in the morning and 3:00 h in the afternoon, using plastic buckets. Prebiotics were dissolved in milk prior to suckling.

### Growth and blood sampling

S2 File shows experimental sequences for weighing and blood samples. The calves were weighed immediately after arriving at the experimental site (day 0) and at the days 15, 28, 45 and 56 (end of experiment). The animals were weighed before the morning suckling using a mechanic scale with capacity of 100 Kg (ValFran–model 602, Sao Paulo, Brazil) and the total body weight gain were calculated as sum of the entire experimental period. Individual blood samples were collected from each animal at 10, 22, 37 and 49 days of the study. The blood samples were taken via jugular venipuncture after local asepsis, using a 10-mL blood collection tube (VacuPlast, Sao Paulo, Brazil) containing no additive for serum dosage of alkaline phosphatase, total ghrelin, leptin, glucose, triglycerides, lactate, creatinine, protein and urea. For this, the blood samples were immediately placed on ice, followed by centrifugation (2,000 × g, 4°C, 15 min) transferred by pipette into 2-mL plastic tubes and stored at -20°C until further biochemical parameters analysis. For serum ghrelin assay, 500 μL of the serum were separately acidified with 50 μL of HCl 1M and stored at −80°C. The serum collected for quantification of leptin was transferred to identified plastic microtubes and stored in a freezer at -80°C.

### Animal harvest and tissue sampling

The calves were shipped 170 km to a Research Institute’s abattoir in Urutai, GO (Instituto Federal Goiano) at the 56^th^ day of the experiment. The final body weight was determined immediately before transporting the cattle. After 10 days of data collection, one calf of MOS group (n = 4) died suddenly of unknown causes, before meeting the criteria of euthanasia. At the end of the experiment all the animals were desensitized by stunning and slaughtered. After the slaughter, the brain was promptly removed from skull and the paraventricular region of the hypothalamus was quickly collected, packed in plastic microtubes, frozen in liquid nitrogen and stored at −80°C to determine the expression of ghrelin receptor. Transverse sections from the medial part of the duodenum and jejunum, as well as fragments from the left cranial rumen sac were collected from all calves. The sample fragments were washed in distilled water and immediately placed in 10% buffered formalin (pH 7.4) for 24 hours. After this time, the tissues were carefully washed in running water and kept in 70% alcohol until the histological procedures were performed.

### Metabolites and hormones

The metabolites were analyzed by commercial kits from Labtest (Lagoa Santa, MG, Brazil), which were read by automatic spectrophotometer (Labmax Plenno Labtest, Lagoa Santa, MG, Brazil). The following kits were used: glucose (1012), lactate (86), creatinine (127), total protein (99), urea (27), alkaline phosphatase (40) and triglycerides (87).

The total serum ghrelin and serum leptin were obtained by the Enyzme Linked Immune Sorbant Assay (ELISA) sandwich technique [20]. In a 96-well polystyrene plate, 100 μL of rat anti-ghrelin antibody (AAU93610 RayBiotech, Norcross, GA, 100 μg/mL) were added per well, then the plate was sealed overnight at 4°C. Rat ghrelin has been used and validated to detect bovine ghrelin in another study [22]. The plate was washed five times using washing buffer (50 mM Tris-HCl containing Tween-20) and blocked with 1% BSA for one hour. After, 20 μL of serum samples and standards were added to the wells and the plate was incubated overnight at 4°C followed by five washes with washing buffer. Sequentially, 100 μL of detection antibody (0.25 μg/mL) (Peprotech) was added to all wells. The plate was covered and incubated for four hours at room temperature in a shaker at a moderate speed. After incubation, the plate was washed five times and 100 μL of enzyme solution (streptavidin-polyHRP80 conjugated peroxidase in PBS) was added and incubated for one hour at room temperature. Finally, the plate was washed five times, and 100 μL/well of Substrate Solution (3, 3’, 5, 5’-tetrametil-benzidina in PBS with H_2_O_2_) were added. After 30 minutes of development, 100 μL of stop solution (0.3M HCl) were added rapidly and the plate was read in a Genios plate reader (Phoenix Research Products, Candler, NC) with an excitation wavelength of 535 nm and an emission filter of 590 nm. The leptin analyzes followed the same methodology used for ghrelin analyze and it was used the anti-human leptin antibody (500-P86 Peprotech, Rocky Hill, NJ). Leptin human homology with bovine leptin was validated in other study [23].

### Histology

The samples from jejunum, duodenum and rumen were processed for 8:45 hours in a tissue processor (Tissue processor: LupeTec PTO5TS; Sao Carlos, Brazil). Fixed samples were embedded in paraffin and serially sectioned into 5 μm thickness slices with an automatic rotator microtome (Leica RM2155, Nussloch, Germany). The sections were stained with hematoxylin and eosin according to the method described previously by [24]. Tissue morphologic characteristics were evaluated under light microscope (Leica DM 2500, Nussloch, Germany) equipped with a video camera QICAM Fast 1394 (QcaptureW, Surrey, BC, Canada) connected to the computer-based image analysis software QImaging (QcaptureW, Surrey, BC, Canada). Duodenum and jejunum tissues were evaluated for villi height and crypt depth (50x of magnification), while rumen tissue was evaluated for rumen papillae length (25x of magnification) [25]. Data from all calves were pooled for procedure analysis.

### Western blot analysis

Quantitative Western blot analysis was used for the detection of bovine ghrelin receptor [26]. The hypothalamus sample was homogenized for extract preparations in 250 μL ice-cold mild lysis buffer, containing 1% Nonidet P-40, 0.05 mol/L NaCl, 0.01 mol/L sodium phosphate (pH 7.2), 2 mmol/L ethylene diamine tetra acetic acid, 50 mmol/L sodium fluoride, 0.2 mmol/L sodium vanadate, and 1 g/mL of aprotinin. The tissue homogenates were sonicated on ice (3 cycles, twice for 10 s) and centrifuged at 20 000 × g for 10 min and supernatants were collected. Protein levels in the homogenates were determined using the Bradford methodology [27].

Sodium dodecyl sulphate-polyacrylamide gel electrophoresis was performed on an equivalent amount of protein samples using precast 10% resolving / 5% stacking Tris-HCl gels (Bio-Rad, Hercules, CA). Separated proteins were transferred to nitrocellulose membrane (Amersham Pharmacia Biotech, Inc., Piscataway, NJ). Membranes were blocked in 5% nonfat milk in TBS buffer containing 0.1% Tween 20 (TBST) for one hour at room temperature. Blocked membranes were incubated in primary antibody specific for mouse ghrelin receptor bs-11529R, (Bioss, Woburn, Massachusetts) at a concentration of 1:1000, in TBST overnight at 4°C. The membranes were washed and probed with horseradish peroxidase-conjugated secondary antibody (Amersham Pharmacia Biotech, Inc., Piscataway, NJ) for one hour at room temperature. Chemiluminescence detection was performed with the Amersham enhanced chemiluminescence detection kit according to the manufacturer’s instructions. To ensure a similar amount of protein in each sample, the membranes were "stripped off", reprobed with GAPDH, developed with horseradish peroxidase-conjugated secondary antibody, and visualized by enhanced chemiluminescence.

### Statistics and analyses

Values are reported as the mean ± the standard error of the mean (SEM). All the variables were examined by one-way ANOVA, considering treatment as factor. When a significant main effect of treatment was observed, the Tukey’s multiple comparisons test was used to compare groups. Data were analyzed using the GraphPad Prism 6 software (GraphPad Software Inc., San Diego, CA, USA), with p < 0.05 considered as significant.

## Results

### Changes in body weight gain and food intake

**Fig 1:**
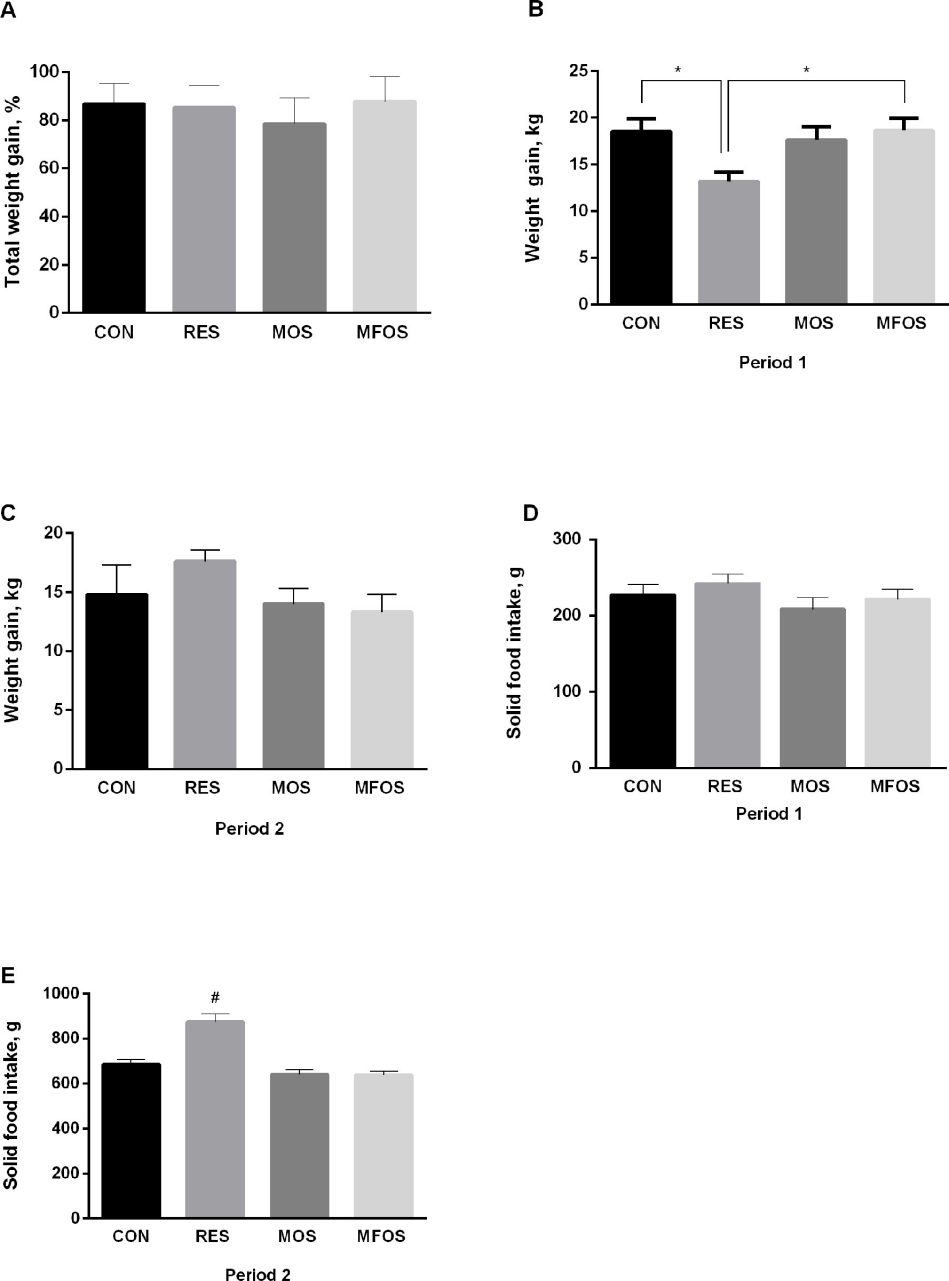
Calves weight gain and solid food intake. The average weight gain during periods 1 (day 1–28) and 2 (day 29–56) (Fig 1B and 1C, respectively), the total weight gain at the end of experiments (Fig 1A) and solid food intake during periods 1 and 2 (Fig 1D and 1E, respectively) of calves underwent milk restriction or supplemented with prebiotics. Data are presented as mean ± the standard error of the mean (SEM). * P<0.05; ^#^ P<0.05 vs. other groups. CON–control calves that received 6 L of milk/day for 56 days. RES–calves received restriction milk (3 L/day) for 28 days (Period 1) followed by 6 L milk/day for 28 days (Period 2). MOS–calves supplemented with 5 g/day of mannanoligosaccharides and same diet of CON animals. MOS calves supplemented with 5 g/day of manan-fructooligosaccharides and same diet of CON animals.

Fig 1 shows the changes in the average weight gain, the total weight gain and solid food intake of calves that underwent milk restriction or supplemented with prebiotics. The average of weight gain during period 1 (Fig 1B) for RES animals was smaller than control (RES:13.18 ± 0.9 Kg; CON: 18.54 ± 1.4 Kg; P<0.05). Such difference (vs. CON) was not seen in MOS and MFOS animals (MOS: 17.63 ± 1.4 Kg; MFOS: 18.64 ± 1.3 Kg). Converse, during period 2 (Fig 1C) there were no differences among groups with regard to body weight gain. At the end of experiments (day 56 = period 1 + period 2) the total weight gain was similar among groups (Fig 1A—CON: 86.79 ± 8.6%; RES: 85.28 ± 9.1%; MOS: 78.56 ± 10.9%; MFOS: 87.81 ± 10.43%, respectively). Fig 1D and 1E show the intake of solid food by treatments during periods 1 and 2, respectively. No difference was found for the consumption in the first 28 days of the experiment, in contrast, in the period 2 RES animals had higher consumption of solid food compared to other animals (RES: 875.2 ± 34.0 g; CON: 686.3 ± 21.0 g; MOS: 640.3 ± 22.8 g; MFOS: 638.4 ± 18.1 g).

### Metabolites and hormones

**Fig 2:**
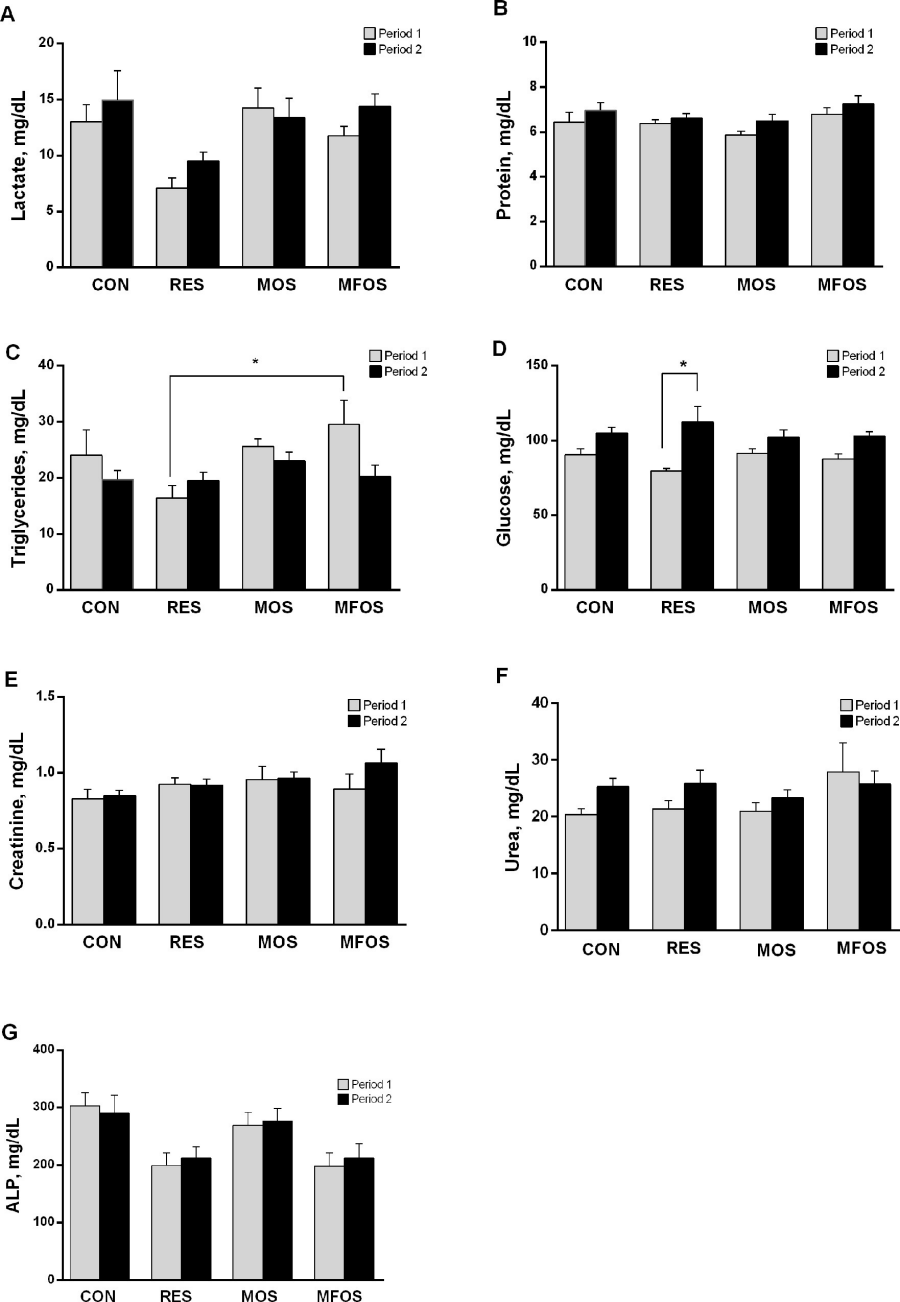
Changes in metabolites of calves underwent milk restriction or supplemented with prebiotics. Serum levels of lactate (Fig 2A), protein (Fig 2B), triglycerides (Fig 2C), glucose (Fig 2D), creatinine (Fig 2E), urea (Fig 2F) and alkaline phosphatase (Fig 2G) of calves fed milk restricted or supplemented with prebiotics during period 1 (gray bars) and period 2 (black bars). Data are presented as mean ± the standard error of the mean (SEM). *P<0,05. CON–control calves that received 6 L of milk/day for 56 days. RES–calves received restriction milk (3 L/day) for 28 days (Period 1) followed by 6 L milk/day for 28 days (Period 2). MOS–calves supplemented with 5 g/day of mannanoligosaccharides and same diet of CON animals. MOS calves supplemented with 5 g/day of manan-fructooligosaccharides and same diet of CON animals.

**Fig 3:**
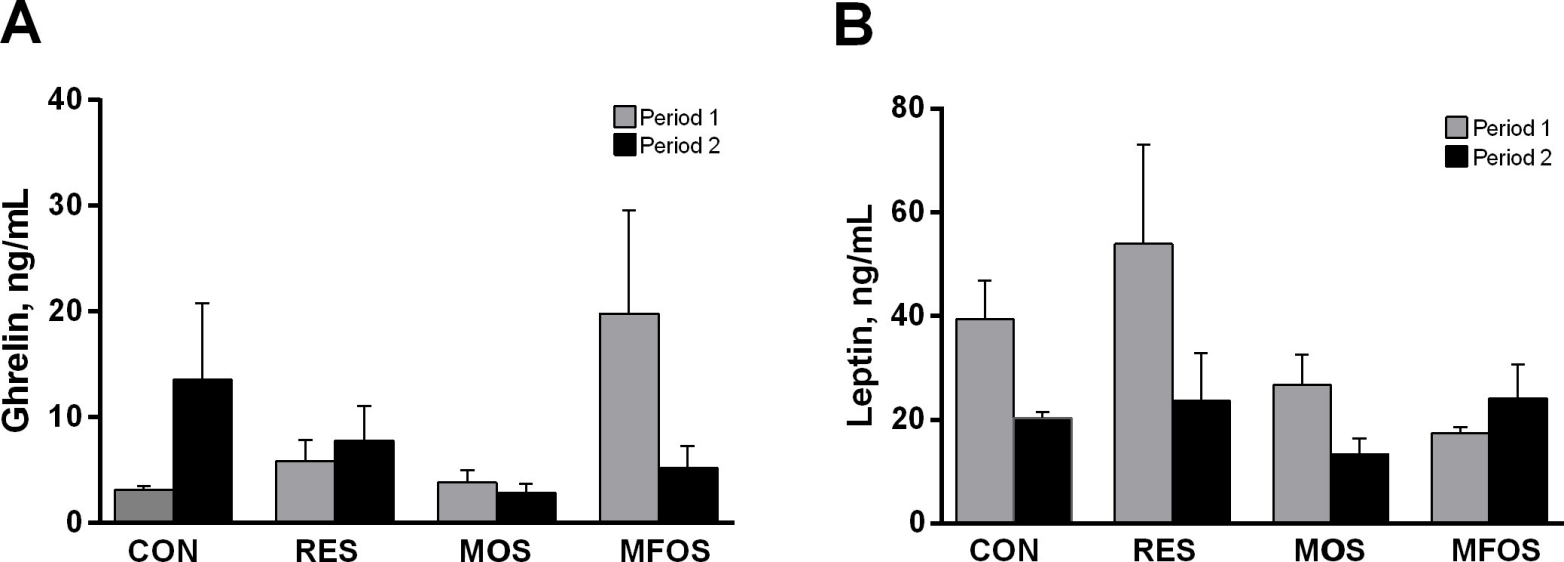
Hormone concentrations of calves underwent milk restriction or supplemented with prebiotics. Ghrelin (Fig 3A) and leptin (Fig 3B) serum levels during period 1 (gray bars) and period 2 (black bars). Data are presented as mean ± the standard error of the mean (SEM). CON–control calves that received 6 L of milk/day for 56 days. RES–calves received restriction milk (3 L/day) for 28 days (Period 1) followed by 6 L milk/day for 28 days (Period 2). MOS–calves supplemented with 5 g/day of mannanoligosaccharides and same diet of CON animals. MOS calves supplemented with 5 g/day of manan-fructooligosaccharides and same diet of CON animals.

The comparison of the changes in metabolites and in hormone concentrations obtained from all groups and treatments are shown in Figs 2 and 3, respectively. There were no differences among groups and periods of treatments regarding to serum levels of lactate (Fig 2A), protein (Fig 2B), creatinine (Fig 2E), urea (Fig 2F) and alkaline phosphatase (Fig 2G). Triglyceride level (Fig 2C) was higher in the MFOS group compared to RES during period 1, no change was found for the other groups in the same period or between groups in period 2. Glucose did not alter between groups in the two periods (Fig 2D), in contrast was lower in the RES animals in period 1 compared to period 2. Restriction (RES) and supplementation (MOS and MFOS) were unable to modify the serum levels of ghrelin (Fig 3A) and leptin (Fig 3B).

### Epithelial growth

**Fig 4:**
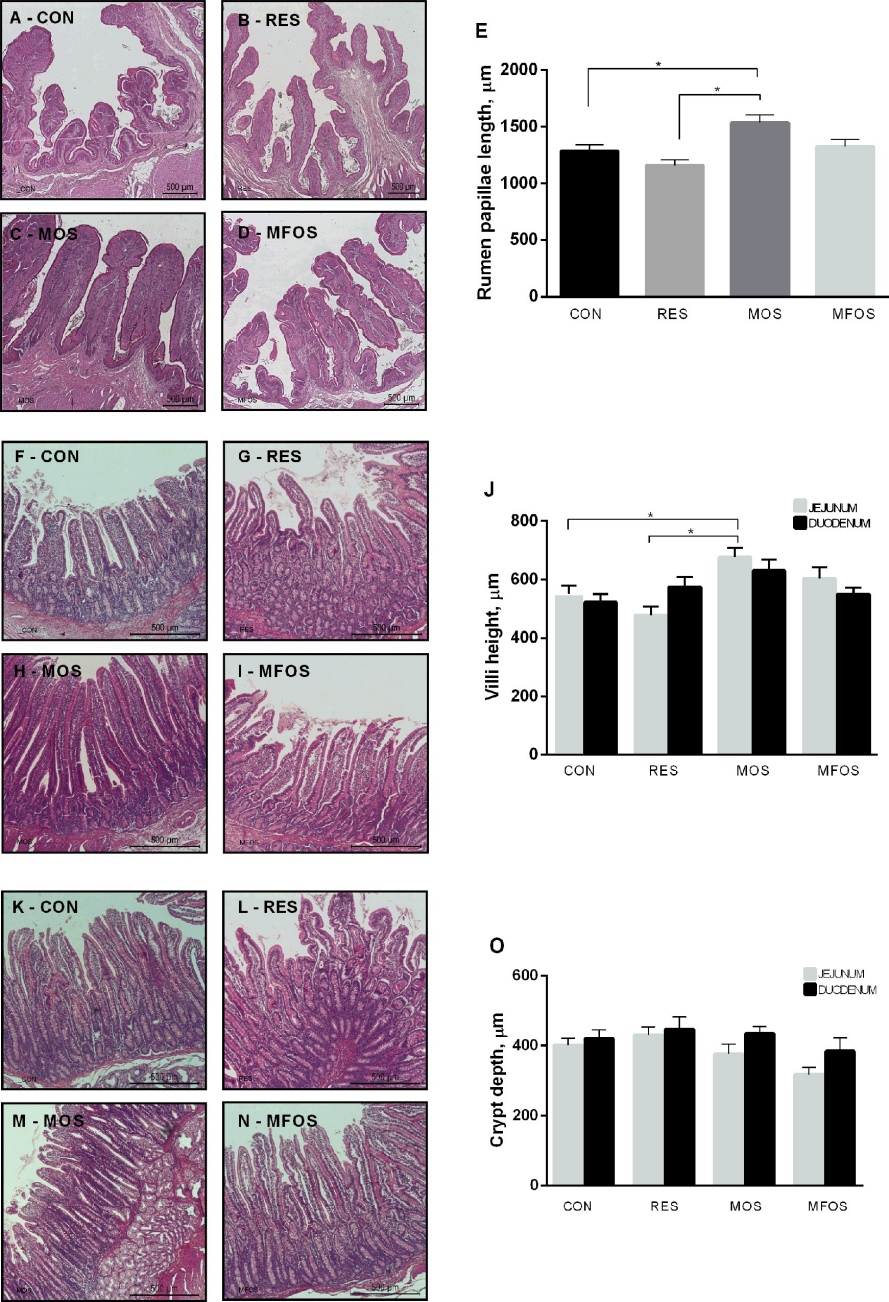
Histomorphometry of ruminal papillae and small intestine mucosa of calves. Representative images of ruminal papillae (Fig 4A–4C), jejunum villi (Fig 4F–4I) and duodenum villi (Fig 4K–4N). Length of ruminal papillae (Fig 4E); height of villi (Fig 4J) of jejunum (gray bars) and duodenum (black bars); Crypt depth (Fig 4O) of jejunum (gray bars) and duodenum (black bars) after 56 days of study of calves that underwent milk restriction or supplemented with prebiotics. Data are presented as mean ± the standard error of the mean (SEM). *P<0,05. CON–control calves that received 6 L of milk/day for 56 days. RES–calves received restriction milk (3 L/day) for 28 days (Period 1) followed by 6 L milk/day for 28 days (Period 2). MOS–calves supplemented with 5 g/day of mannanoligosaccharides and same diet of CON animals. MOS calves supplemented with 5 g/day of manan-fructooligosaccharides and same diet of CON animals.

Fig 4 shows the effect of both milk restriction and of prebiotics supplementation in rumen papillae and villus of the small intestine of calves. The length of the rumen papillae (Fig 4E) was greater (P < 0.001) in MOS (1536.04 ± 67.10 μm) compared to CON (1284.33 ± 58.92 μm) and RES (1159.47 ±4 6.87 μm) group. However, the supplementation with manan-fructooligosaccharides (MFOS group) did not affect the papilla size (1323.95 ± 66.34 μm). Concerning the villus height, MOS supplementation significantly increased jejunum villus height (Fig 4J) (676.21 ± 32.59 μm) ratio (P < 0.001) compare with CON (551.59 ± 27.58 μm) and RES (478.08 ± 28.64 μm) groups, but MOS group was not different from MFOS (603.29 ± 39.04 μm) (Fig 4J). No statistical differences were observed in duodenal villi height among groups (Fig 4J) (P > 0.05). At the same time, no differences were found in the crypt depth in both duodenum and jejune among groups (P > 0.05) (Fig 4O).

### Expression of ghrelin receptor in hypothalamus

**Fig 5:**
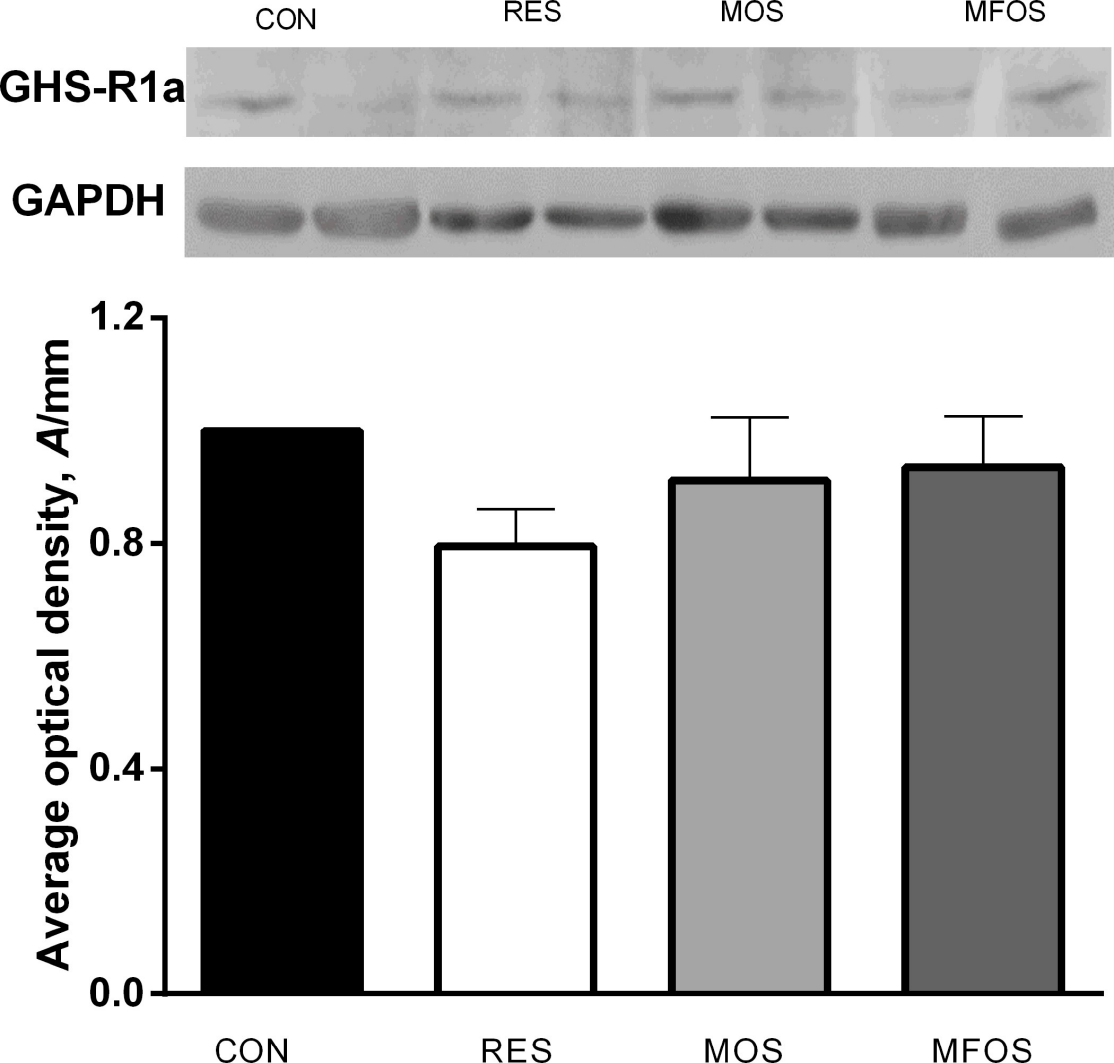
Gene expression of ghrelin in hypothalamus of calves. Expression of ghrelin receptor (GHS-R1a) in the paraventricular region of the hypothalamus of calves underwent milk restriction or supplemented with prebiotics. Values are presented as mean ± standard error. CON–control calves that received 6 L of milk/day for 56 days. RES–calves received restriction milk (3 L/day) for 28 days (Period 1) followed by 6 L milk/day for 28 days (Period 2). MOS–calves supplemented with 5 g/day of mannanoligosaccharides and same diet of CON animals. MOS calves supplemented with 5 g/day of manan-fructooligosaccharides and same diet of CON animals.

The expression of GHS-R1a in the hypothalamus is presented in Fig 5 and was not different among control and treatments (RES, MOS and MFOS) (P > 0.05).

## Discussion

Our main findings were: i) Milk restriction caused lower weight gain during restriction period; ii) After milk restriction, calves had greater solid feed consumption and compensatory gain weight; iii) Mannanoligosaccharides supplementation improved rumen and intestinal mucosa development; iv) Milk restriction or prebiotics supplementation did not alter serum ghrelin and leptin and ghrelin receptor in the hypothalamus. The compensatory gain observed for RES group in this study was initially proposed as the fast weight gain after a period of feed restriction [28, 29]. The CG results may vary among individuals, despite the literature considers that the compensatory gain may be improved if the duration of growth restriction is short and not so severe [17]. In our study, the methodological choice of dietary restriction was not severe enough to change calves solid feed intake during the period 1. The consumption of concentrate and roughage is very small in the first two weeks of calves life [30] and cannot immediately compensate the reduction in milk intake, which explains the reducing in the body weight gain observed in period 1 of RES group. In prior reports, faster weight gain of restricted and re-alimented periods is firstly associated with the recovery of metabolic activity and thereafter with the development of the liver and small intestine in the second period [6]. However, in our experiment, milk restriction for 28 days did not modify villi and crypt development in duodenum and jejunum, as observed in RES calves. Furthermore, the GC responses may be attributed to the restoration of the amount of milk (6 L/day) and the increase in solid food intake in RES group, during period 2, when compared to the CON, MOS and MFOS groups. Curtis and colleagues [31] showed that 3-week-old calves submitted to milk substitute restriction increased solid food intake, which coincided with the increase in growth of these animals when compared to the group fed *ad libitum*.

It has been shown that solid food intake increases the size of the rumen as well as the dimensions of rumen papillae [32,33]. Although our results showed that the RES group had higher solid food intake in the period 2, there were no differences in rumen papillae length. Intriguingly, however, supplementation with MOS dissolved in milk resulted in increasing of rumen papillae length papillae, in spite of the lack of difference in dry matter intake when compared to CON group. Papilla measures may be the main developmental variable of rumen epithelium, being the gold standard method to detect the influence of treatment on rumen development [24]. The type and composition of the liquid food may indirectly affect the length of the ruminal papillae by modulating the development of the small intestine [34].

The final products of the prebiotics fermentation are the production of volatile fatty acids (VFA’s) [35]. In the present study, the manan-oligosaccharide supplementation (MOS group) improved the intestinal and ruminal tissue, probably due to promoting a boosted energy availability, thus favoring a better development and renewal of cells in intestinal and ruminal tissue. The increase in available VFA’s in the intestinal lumen is associated with increased serum levels of glucagon like peptide (GLP) and consequent increasing in ruminal papillae development [34,36]. On the other hand, our results showed that the supplementation of manan-frutoligosaccharide (MFOS group) did not alter the ruminal tissue growth, which corroborates with an earlier study that reported no significant increase in height of the papillae when prebiotics were given as supplement to suckling calves [4].

The current study confirmed that a prebiotic supplementation boost on intestinal development, as seen in the higher villus height of the MOS group. The VFA butyrate is used as an energy source by enterocytes, stimulates cell proliferation, differentiation and improves intestinal barrier function which leads to higher villus and depth crypts [2, 7, 8, 9]. Changes in villus structure obtained with prebiotic supplementation result in a greater capacity of absorption of nutrients by the intestine. However, the improved development of jejunal villus did not reflect the performance of the animals in this currently study. Literature comprises conflicting data on the effect of prebiotics use and animal performance in the first months of life [9, 11, 12, 13, 14]. Current findings of our study indicate that during weaning, calves must transit from a milk- based diet to one based on solid feed intake, in which MOS supplementation may prepare the calves for faster adaptation during weaning.

We did not find changes in serum creatinine, total protein, urea and alkaline phosphatase levels among the treatments and experimental periods (Fig 2). In fact, little variations were expected, concerning some metabolites parameters, since at 5 days of age creatinine levels are already close to those observed in adult animals [37], and the previous study did not find differences in plasma urea between calves fed with *ad libitum* milk or conventional system [38]. On the other hand, we found that serum triglyceride concentration was significantly lower in the RES group in period 1 compared to the MFOS group. It has been previously reported that during the food restriction considerable levels of triglycerides can be used to supply the energy expenditure [39,40], which corroborate this result. Also, the lower glucose level in period 1 than in the period 2 in RES group support the hypothesis that triglycerides are being used as an energy source. The lack of glucose changes among treatments in both periods suggests that the restriction allows a compensatory change in the synthesis and/or use of glucose maintaining a normal glycemia. In fact, literature reports normal pre-prandial glucose levels (70 to 120 mg/dL) in suckling calves [41].

In cattle, there is an increase in ghrelin and a decrease in leptin levels just before meals in response to fasting and decreased ruminal filling [42]. In the current study, serum concentrations of total ghrelin and leptin, as measured once in each experimental period, did not differ among treatments. Miura and coworkers showed a difference in pre and post-prandial plasma ghrelin concentrations of approximately 90 ng/mL in adult cows and much less variation in young calves [22]. Several factors, including body size and/or composition, feeding frequency and diet composition or amount supplied may affect ghrelin responsiveness among experiments [43,44,45]. In our study, solid feed was available *ad libitum*, and this could interfere in the ghrelin and leptin results.

To further investigate the mechanisms underlying the faster gastrointestinal development, we measured the expression of ghrelin receptor by western blotting assay in the hypothalamus at the end of the experiment. The hypothalamus plays an important role in regulating energy homeostasis and food seeking and intake behaviors in several animal species [46,47,48]. Ghrelin receptor (GHS-R) is expressed throughout the hypothalamus, mainly in the arcuate nucleus [47,48]. Our results showed that GHS-R1a levels in hypothalamus were not affected by restriction (negative energy balance), re-alimented period (positive energy balance) or prebiotic supplementation. This result is strikingly different from that observed in rats (a non-ruminant species), in which fasting periods significantly increased the level of GHS-R1a mRNA in the hypothalamus [49]. On the other hand, our data on cattle corroborate with those from Chen and colleagues [50] that also did not find difference in the expression of ghrelin receptors in the hypothalamus of broilers with 30 days of age submitted to different feeding strategies.

## Conclusions

Calves feeding milk restricted in the first 4 weeks of life had a smaller gain of weight during restriction period followed by compensatory gain of weight and increasing in solid food intake during the re-alimented period. A prebiotic supplementation did not reflect better performance of the animals, although it had improved the development of intestinal and ruminal mucosa. However, these alterations presented by the different treatments did not alter the metabolic, hormonal parameters and the expression of GHS-R1a in the hypothalamus. In summary, supplementation of mannan-oligosaccharide was efficient to accelerate the development of the gastrointestinal tract and prepares the animals for the weaning period.

## Supporting information

S1 File Ethics committee on animal use (CEUA) protocol. The approved protocol by Ethics Committee on Animal Use (CEUA) from Federal University of Goias (UFG)–Protocol 017/16.
(PDF)

S2 File Experimental sequence. Experimental sequences for weighing and blood samples.
(PDF)

S3 File Data. The data sheet provided for this study includes all relevant information of the study. The sheet has one horizontal line for each individual calf in the experiment and each calf has its own identification number (‘ear tag number’). All subsequent columns have an explanation line on the top of the sheet and whenever data is not available for the particular calf this is marked with a ‘.’.
(XLSX)
