# Supplementary material for: Milk restriction or oligosaccharide supplementation in calves improves compensatory gain and digestive tract development without changing hormone levels
Source: PLoS One. 2019 Mar 28;14(3):e0214626. doi: 10.1371/journal.pone.0214626 (PMC6438680; doi:10.1371/journal.pone.0214626)
Supplement: S1 File — The approved protocol by Ethics Committee on Animal Use (CEUA) from Federal University of Goias (UFG)–Protocol 017/16. (PDF) [file pone.0214626.s001.pdf]

MINISTÉRIO DA EDUCAÇÃO
UNIVERSIDADE FEDERAL DE GOIÁS
PRÓ-REITORIA DE PESQUISA E INOVAÇÃO
COMISSÃO DE ÉTICA NO USO DE ANIMAIS/CEUA

UFG

Goiânia, 18 de abril de 2016.

PARECER CONSUBSTANCIADO REFERENTE AO ATENDIMENTO DE PENDÊNCIA DO PROTOCOLO Nº. 017/16

I. IDENTIFICAÇÃO:

1. **Título do projeto**: Manejo Alimentar e uso de MOS no desenvolvimento do trato digestivo de bezerros
2. **Pesquisador Responsável:** Reginaldo Nassar Ferreira
3. **Unidade/Órgão do pesquisador:** Departamento de Ciência Biológicas – Fisiologia-UFG
4. **Pesquisadores Participantes:** Natália Alves Costa; Carlos Henrique Castro; Aline Priscila Pansani, Diego Basile Colugnati
5. **Unidade onde será realizado:** Escola de Veterinária e Zootecnia -UFG
6. **Data de apresentação do protocolo a CEUA: 11/03/2016**
7. **Data de Atendimento das Pendências: 07/04/2016**

II - Parecer da CEUA:

As pendências foram atendidas, segundo carta de atendimento às pendências.

Informamos que a *Comissão de Ética no Uso de Animais/CEUA* da Universidade Federal de Goiás, após análise das adequações solicitadas, **Aprovou,** o projeto acima referido e o mesmo foi considerado em acordo com os princípios éticos vigentes.
Reiteramos a importância deste Parecer Consubstanciado, e lembramos que o(a) pesquisador(a) responsável deverá encaminhar à CEUA-PRPI-UFG o Relatório Final baseado na conclusão do estudo e na incidência de publicações decorrentes deste, de acordo com o disposto na Lei nº. 11.794 de 08/10/2008, e Resolução Normativa nº. 01, de 09/07/2010 do Conselho Nacional de Controle de Experimentação Animal-CONCEA. O prazo para entrega do Relatório é de até 30 dias após o encerramento da pesquisa, prevista para conclusão em março de 2018.

III - Data da reunião: 18/04/2016

Dra. Renata Mazaro e Costa
Coordenadora da CEUA/PRPI/UFG

Comissão de Ética no Uso de Animais/CEUA
Pró-Reitoria de Pesquisa e Inovação/PRPI-UFG, Caixa Postal: 131, Prédio da Reitoria, Piso 1, Campus Samambaia (Campus II) - CEP:74001-970, Goiânia – Goiás, Fone: (55-62) 3521-1876.
Email: ceua.ufg@gmail.com
