# Supplementary figures and images for: Milk restriction or oligosaccharide supplementation in calves improves compensatory gain and digestive tract development without changing hormone levels
Source: PLoS One. 2019 Mar 28;14(3):e0214626. doi: 10.1371/journal.pone.0214626 (PMC6438680; doi:10.1371/journal.pone.0214626)

## Supporting information 2. Experimental sequences for weighing and blood samples

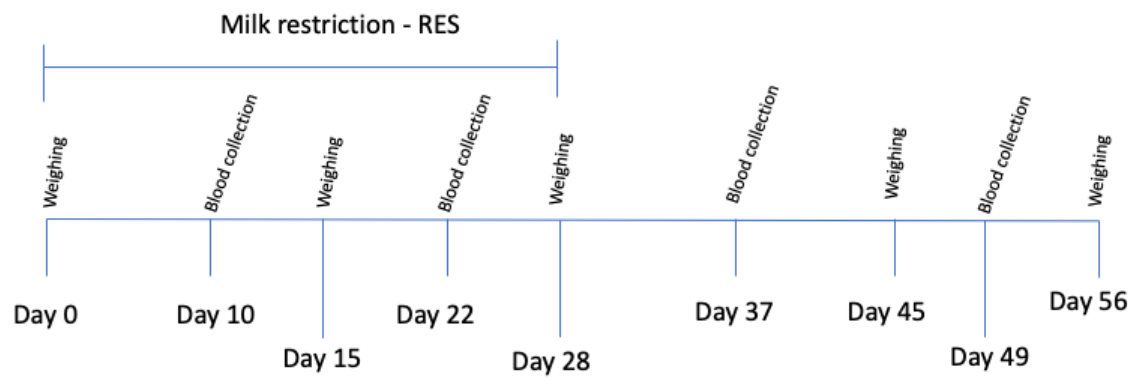

Supplement: S2 File — Experimental sequences for weighing and blood samples. (PDF) [file pone.0214626.s002.pdf]
